# Radiographic optimization of the lateral position of the knee joint aided by CT images and the maximum intensity projection technique

**DOI:** 10.1186/s13018-021-02740-8

**Published:** 2021-10-09

**Authors:** Shiming Wang, Zhibo Xiao, Yunfeng Lu, Zhiwei Zhang, Fajin Lv

**Affiliations:** Department of Radiology, the First Affiliated Hospital of Chongqing Medical University, Number 1, Youyi Road, Yuzhong District, Chongqing City, China

**Keywords:** Maximum intensity projection, Knee joint, Standard lateral, Radiography

## Abstract

**Background:**

Standard lateral knee-joint X-ray images are crucial for the accurate diagnosis and treatment of many knee-joint-related conditions. However, it is difficult to obtain standard lateral knee-joint X-ray images in the current knee-joint lateral radiography position.

**Purpose:**

To optimize the lateral position of knee joint for radiography aided by computed tomography (CT) images and the maximum intensity projection technique.

**Materials and methods:**

One hundred cases of anteroposterior and lateral radiographs of knee joints were included. Of these, 50 cases were for lateral radiography in conventional position, and the other 50 cases were for lateral radiography in optimized position. The optimized position was acquired by a retrospective analysis of one hundred cases of knee-joint CT images. The quality of the X-ray images in optimized group was compared with those in conventional group. The data were statistically analyzed using the Mann–Whitney *U* test.

**Results:**

There were differences in the optimized position between males and females. The posterior condyles of the femoral epiphysis in optimized group were in perfect superimposition for most patients. However, the ones in conventional group were not. The average quality score of the lateral knee-joint X-ray images in optimized position was 3.76 ± 0.98, which is much higher than the 1.84 ± 1.15 score in conventional position. Moreover, the difference in the average quality score was statistically significant (*P* < 0.05).

**Conclusion:**

Optimization of the lateral position of knee joint for radiography is possible with the aid of CT images and the maximum intensity projection technique.

## Introduction

The knee joint is the largest and most complex joint in the human body. It is also one of the joints with the highest proportion of injuries and is subject to the highest number of clinical limb examinations in modern society [1–4]. Radiography is the most common examination technique, and it remains the preferred assessment method for most knee-joint injuries/conditions and constitutes an essential preoperative examination in most knee surgery cases [5–7].

Acquiring radiograph images from the anteroposterior and lateral positions is the most common positions for the knee joint [7]. The lateral position is more important for many knee-joint diagnoses [5–7]. A lateral knee-joint X-ray image should meet the following requirements: (1) The knee joint is in the middle of the image, and there is sufficient overlap between the medial and lateral femoral condyles. (2) The patella is clearly shown in the lateral position with a clear femoral space. (3) There is minimal overlap between the platforms of the tibia and femur. (4) The texture of the knee bones is clear, and the surrounding soft tissue is recognizable [8]. Of those requirements, (1) is the most crucial reference standard for assessing the quality of the lateral image of the knee joint. It is also the underlying image for the clinical diagnosis of patella instability and stunted patellofemoral block, and the measurement and evaluation of the size of the tissue structures during preoperative knee replacement assessments [9–11]. However, in most humans, there is no generic medial and lateral femoral condyles size [12]. Therefore, it is difficult to achieve a complete overlap of the medial and lateral femoral condyles on a lateral X-ray image of the knee joint. Consequently, the international consensus from orthopedic surgeons on the diagnosis of knee-joint-related conditions (such as patellofemoral joint dislocation or dysplasia) mainly relies on standard lateral knee images that have perfect superimposition of the posterior condyles of the distal femoral epiphysis [5–10].

However, in the case of lateral knee-joint X-ray images that were acquired using the current knee lateral radiography position, the perfect superimposition of the medial and lateral femoral posterior condyles [5] is problematic. The X-ray images cannot suitably meet the imaging requirements for the clinical diagnosis of patella instability and stunted patellofemoral block. The position also hinders the measurement and evaluation of tissue structure size during preoperative knee replacement. Henceforth, further optimization of the conventional lateral knee-joint radiography position is required.

The standard lateral knee-joint X-ray image, which includes the perfect superimposition of the posterior condyles of the distal femoral epiphysis, is required for realizing the clinical diagnosis of patella instability, patellar synovial dysplasia, and for the measurement and evaluation of tissue structures before knee replacement [8–10].

As a commonly used CT image post-processing technique, the maximum intensity projection (MIP) technique can project a series of continuous knee-joint CT images into a two-dimensional image that is similar to the knee-joint X-ray images. The image can be simultaneously rotated at any angle using the MIP technique in order to achieve the specific angle and position required, such as the standard lateral position for observation. However, the patients’ effective radiation dose equivalent value is too large (about five times that of conventional radiography) during CT images acquisition, upon which the MIP technique relies. In addition, inspection fees are high. Furthermore, conventional CT technology cannot achieve knee-joint examination under clinical weight-bearing positions [5]. Therefore, standard lateral knee-joint images are still required. In this study, existing knee-joint CT images were used to retrospectively analyze the relative anatomical location of the medial and lateral condyles of the femur. Additionally, the MIP technique was applied to guide the optimization of the lateral knee-joint’s radiography position.

## Methods

### General data

In authors’ hospital, anteroposterior and lateral radiograph knee-joint images acquired between June 1, 2020, and June 25, 2020, were included in this study. Cases of severe knee-joint arthritis, noticeable knee-joint distortion, or dislocation, and with a history of knee-joint surgery were excluded.

### Examination methods

All cases were randomly divided into two groups. One group would be placed in the conventional position for lateral knee-joint radiography, and the other group was placed in an optimized position for lateral knee-joint radiography. The conventional position was that the femur and the tibiofibular was at an angle of approximately 120$^\circ$, the standard middle sagittal plane of the knee joint was parallel to the detector, and the centerline of X-ray was incident vertically through the center of the patellofemoral joint. The optimized position, resulted from the retrospective analysis of the knee-joint CT images, was described in more detail in the following relevant section ‘the optimal radiography angle for the standard lateral knee joint.’ All the cases in the two groups applied the same anteroposterior position for radiography. All the knee-joint X-ray images were acquired by a Ruike DR 3500, the source to image-receptor distance (SID) was 120 cm, and the exposure parameter was 60 kV at 8 mA.

### Determination of the standard lateral radiography position for knee joint

One hundred cases of knee-joint CT images initially acquired in our hospital from June 2018 to May 2019 were retrospectively analyzed. They were divided into mate and female groups, and the optimal inward rotation and outward tilt angles of the left and right knee joints were analyzed.

All CT images for retrospective analysis were acquired by spiral scanning using a Siemens SOMATOM Perverse 128 row of VCT machine. The scan parameter was 130 kV at 200 mA, the pitch was 0.9:1, and the field of view (FOV) was 14 to 15 cm. The standard inspection position was used in all cases. In this position, the long axis of the examined knee joint was parallel to the long axis of the inspection bed, the patient’s feet were faced upward, and the midline of the foot (formed by the third metatarsal bone and the middle point of the posterior edge of the calcaneus) was perpendicular to the surface of the inspection bed. The original thickness of the images was 5 mm, and the reconstruction matrix was 512 × 512. The original data were reconstructed to a 0.6 mm image thickness. The MIP technique was the most commonly used recombination method.

**Fig. 1:**
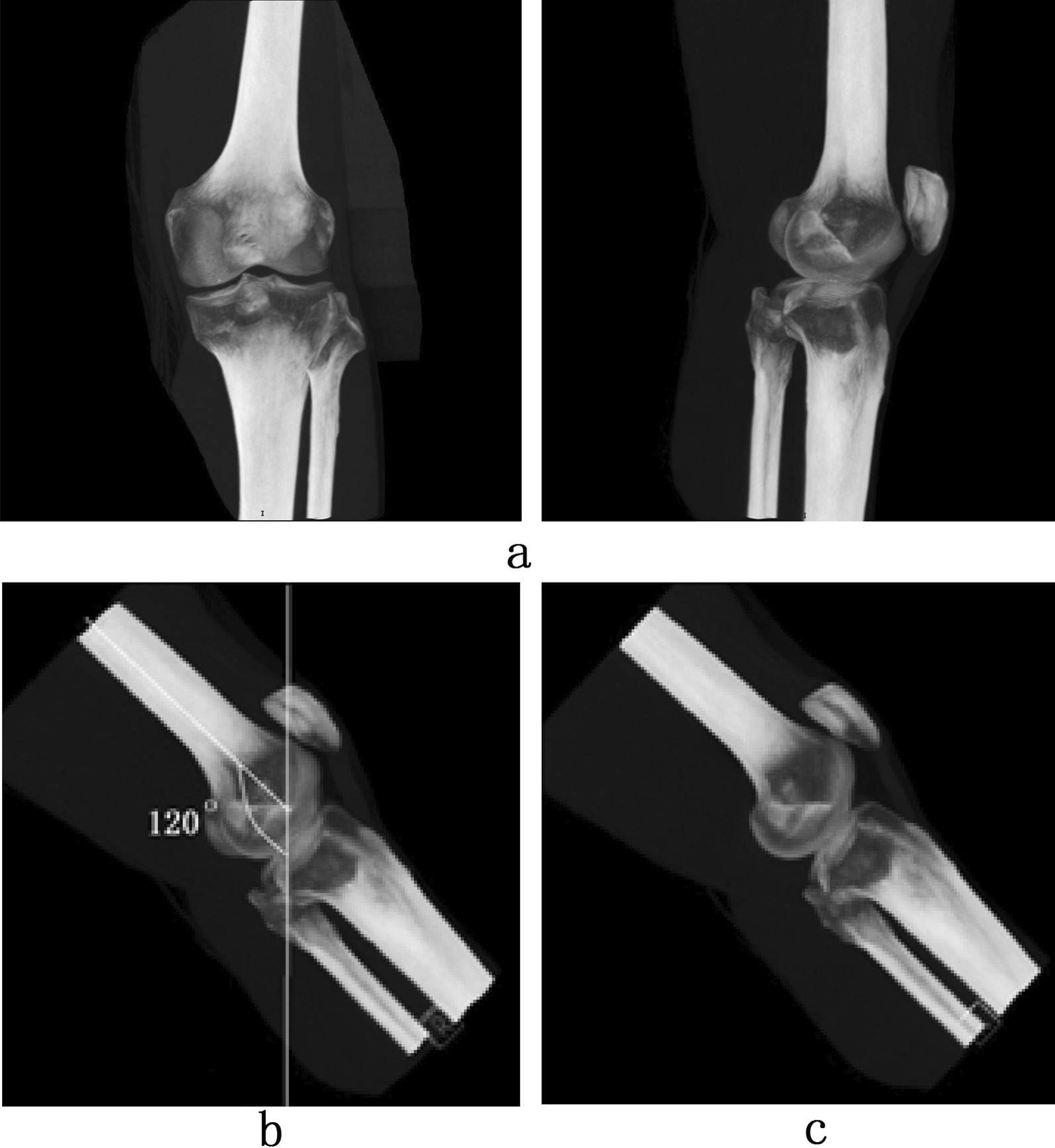
Schematic of the analysis on the optimal projection angle for the standard lateral knee joint in an MIP image (**a**) anteroposterior and lateral knee-joint MIP images, (**b**) reference image of lateral knee joint, (**c**) standard lateral knee-joint image

The specific measurement process is shown in Fig. 1. The knee-joint image for each case was first processed using the MIP technique on the syngo MMWP VE40C image post-processing workstation. The workstation produced images similar to the anteroposterior and lateral knee-joint X-ray images, respectively (Fig. 1a). Then, the lateral image was adjusted to the required angle while keeping the tibia at the front and the fibula at the back. The proximal knee was tilted at 60° to the dorsal side to allow the distal femur and the medial and lateral femoral condyles to align with the knee in the conventional lateral knee-joint X-ray image [8, 11]; the angle between the distal femur and the vertical line of the ground in the down direction was 120° (Fig. 1b). The anatomical positional relationship of the femur’s medial and lateral condyles was observed on the anteroposterior and lateral images, using it as a reference image.

According to the anatomical positional relationship of the upper/lower directions and the front/rear directions, the reference image was adjusted to a specific inward rotation (angle A) and an outward tilt (angle B) to achieve a true standard lateral knee-joint image of the perfectly imposed posterior condyles of the distal femoral epiphysis (Fig. 1c). During the adjustment process, angles A and B were observed and analyzed by a radiologist with five years’ experience and a radiographer with more than five years’ experience. The final angle values were confirmed by the clinicians’ consensus and were allocated to group A and group B, respectively.

### Clinical practice and comparative analysis

The acquired adjustment direction and angles for the standard lateral knee joints were applied in clinical lateral knee-joint radiography. The outward tilt angle was determined by the anatomical positional relationship of the upper end of the femur’s medial and lateral posterior condyles on the anteroposterior images. The inward rotation angle was determined by touch to define the anatomical positional relationship between the lower end of the femur’s medial and lateral posterior condyles. The captured lateral knee-joint X-ray images by optimized position were compared with ones captured by the conventional lateral knee-joint radiography position. The image quality scores of the two groups were rated by the radiologist and the radiographer mentioned above. The final scores were confirmed by their accord and were allocated to group O (optimized group) and group T (conventional group), respectively. The scoring standard was (1) five points for a perfect superimposition of the posterior condyles of the distal femoral epiphysis and a clear presentation of patellofemoral clearance, (2) three points for the perfect superimposition of the posterior condyles of the distal femoral epiphysis or the clear presentation of patellofemoral clearance, and (3) one point for the poor superimposition of the posterior condyles of the distal femoral epiphysis and an unclear presentation of patellofemoral clearance.

### Statistical analysis

IBM SPSS 22.0 statistics software was used for the statistical analysis. The normal distribution data were represented by the mean ± standard deviation (*X* ± *S*), and the abnormal distribution data were represented by median and quartile values. When comparing image quality scores of the knee-joint X-ray images in the O and T groups, and when comparing the optimal deflection angles for the projection of the knee joint between males and females or between left and right, independent sample *t* tests were used if the data were normal and if the variances were homogeneous. The Mann–Whitney *U* test was applied if the data were not normally distributed or showed variance.

The study was approved by the local ethics committee (2020-082-2).

## Results

### Baseline data

A total of one hundred anteroposterior and lateral knee-joint radiographs were included in this study, with an average radiation dose of 0.04 mSv. Among them, there were fifty cases in the optimized group, and the other fifty cases were in the conventional group.

### The optimal radiography angle for the standard lateral knee joint

A retrospective analysis was performed on one hundred cases of knee-joint CT images, excluding five cases with noticeable knee-joint distortion or knee-joint prosthesis replacement, ninety-five cases remained, with an average radiation dose of 0.21 millisievert (mSv).

It was found that the upper end of the femur’s lateral posterior condyles was often higher than the femur’s medial posterior condyles. Therefore, the knee joint should be tilted outward by some angles (angle B) based on the conventional lateral position to overlap the upper end of the femur’s medial and lateral posterior condyles on the lateral knee-joint images. Also, it was found that the lower end of the femur’s medial posterior condyles was often in front of the ones of the femur’s lateral posterior condyles, so the knee joint should be slightly rotated inward by some angles (angle A) based on the conventional lateral position to make the femur’s medial and lateral posterior condyles in perfect superimposition.

The A and B groups demonstrated approximate normal distribution, so the average numbers of groups within the two groups were compared by an independent *t* test, respectively. The results showed no significant difference between the optimal intorsion angle of the left and right knee joints (*P* > 0.05). Further, no significant difference between the optimal extraversion angle of the left and right knee joints was found (*P* > 0.05). Therefore, it was confirmed that there was no significant difference between the optimal projection deflection angles of the left and right knee joints. Additionally,  as summarized in Table 1, there was no significant difference between the optimal intorsion angles of the knee joints in male and female patients (*P* > 0.05), although there was a significant difference in the optimal extraversion angle (*P* < 0.05). It was concluded that based on the conventional lateral position, the radiograph of the standard lateral knee joint in males was an inward rotation of − 3.61° ± 6.91° and outward tilt of 2.65° ± 4.04°, while the female knee joint was inwardly rotated at − 3.61° ± 6.91° and outwardly tilted at 0.38° ± 3.06°.

**Table 1:** Optimal male and female intorsion and extraversion angles

	Male(°)	Female(°)	*P*	Final angle(°)
A (optimal intorsion angle)	− 2.03 ± 5.37	− 4.62 ± 7.61	0.074	− 3.61 ± 6.91
B (optimal extraversion angle)	2.65 ± 4.04	0.38 ± 3.06	0.003	Male: 2.65 ± 4.04 Female: 0.38 ± 3.06

### The contrast of lateral knee-joint image quality in optimized and conventional position groups

**Fig. 2:**
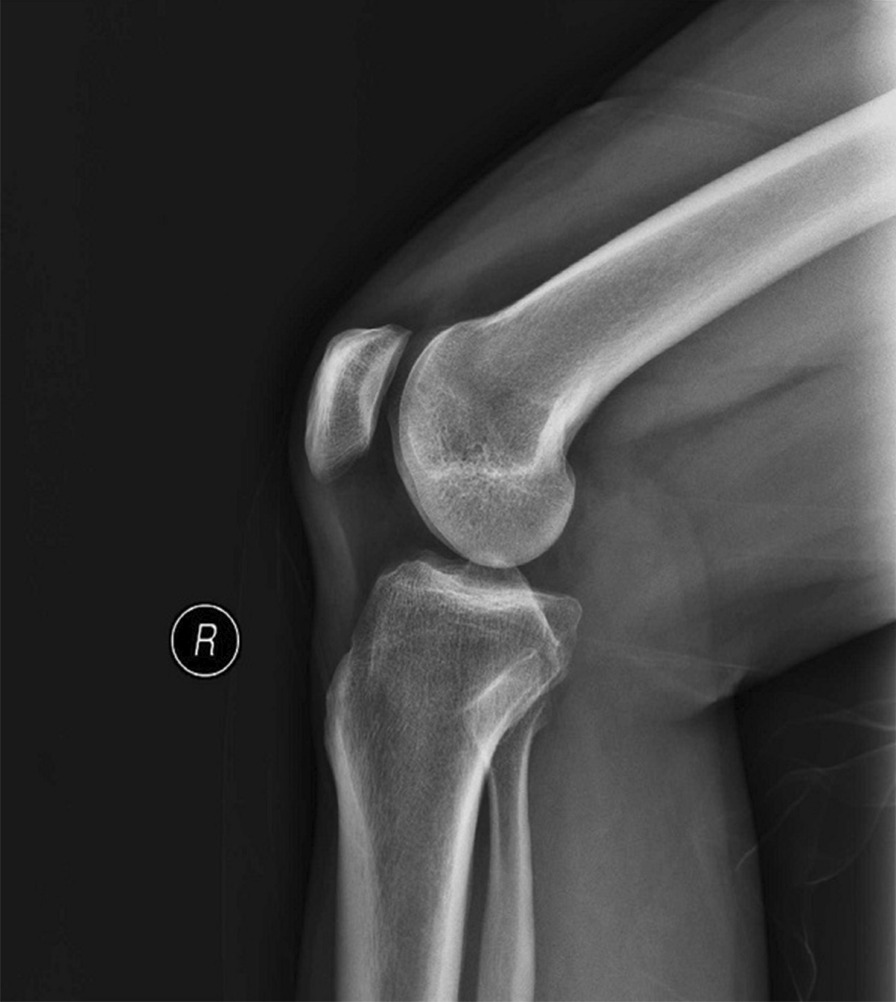
Radiograph of lateral knee joint captured in optimized position

**Fig. 3:**
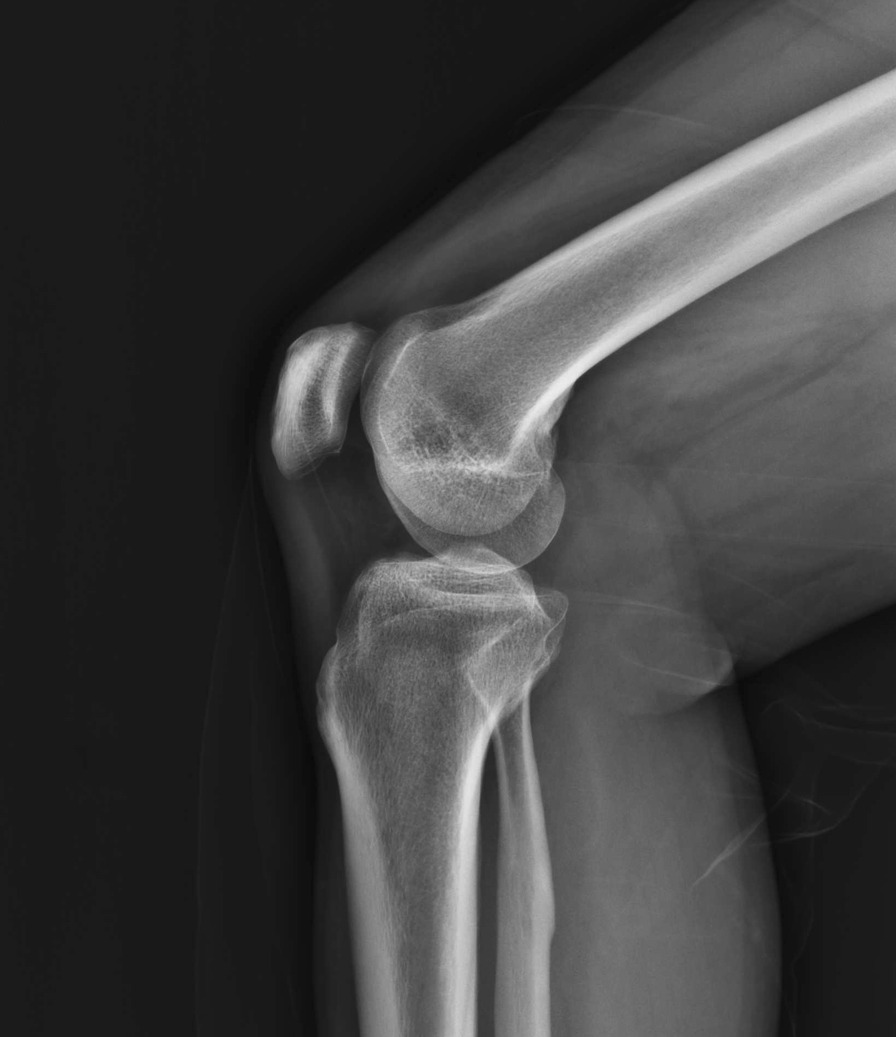
Radiograph of lateral knee joint captured in conventional position

The optimized radiography position was applied in clinical lateral knee-joint radiography. As illustrated in Table 2, the image quality of the X-ray images (Fig. 2) was compared with the X-ray images that were captured using the conventional position (Fig. 3). The posterior condyles of the femoral epiphysis in optimized groups were in perfect superimposition for most patients. However, the ones in conventional groups were not in perfect superimposition. The quality of the lateral knee-joint X-ray images captured in the new optimized position was generally higher than the ones captured in the conventional position. The results confirmed that the image quality score of the O group using the optimized position was 3.76 ± 0.98, which was significantly higher than that obtained with the conventional position (1.84 ± 1.15). The difference between the two groups was statistically significant (*P* < 0.05).

**Table 2:** Contrast of image quality between optimized group (O) and conventional group (T)

	Average image quality score
Group T	1.84 ± 1.15
Group O	3.76 ± 0.98
*P* value	0

## Discussion

The diagnosis and evaluation of knee-joint conditions often require standard X-ray and CT images [12–18]. The mean effective radiation dose equivalent value (0.04 mSv) for the acquisition of knee-joint X-ray images is significantly 81% lower than that of CT (0.21 mSv). The radiographer can also perform radiographs of the knee joint in the weight-bearing position, something that is very important for the subsequent clinical diagnosis and evaluation but is impossible to perform with the conventional CT technique [5]. Therefore, the anteroposterior and lateral knee-joint X-ray images captured in the weight-bearing position are essential. For the knee-joint anteroposterior radiograph, whether in decubitus or standing position, the standard anteroposterior X-ray images can be easily obtained by using the position where the knee joint is straight, the foot is neutral, and the X-ray centerline is incident vertically to the lower edge of the patella [19]. Meanwhile, it is difficult to capture a standard lateral X-ray image with a perfect superimposition of the femoral condyles for the knee-joint lateral radiograph because the size of the medial and lateral femoral condyles of the knee joint is not equal among individuals [20]. However, the standard lateral knee-joint X-ray image is important for the clinical diagnosis of patella instability and stunted patellofemoral block and the measurement and evaluation of tissue structure size during preoperative knee replacement assessments [21]. Especially, the international orthopedic surgeon’s diagnosis of knee-joint conditions (such as patella dislocation and trochlear patella dysplasia) mainly depends on the standard lateral X-ray image in which the posterior condyles of the distal femoral epiphysis are in perfect superimposition [5, 9]. This study confirmed that the medial posterior condyle of the distal femoral epiphysis was lower and more forward than the lateral posterior condyle. The knee joint was inwardly rotated and outwardly tilted to a required degree based on the conventional lateral knee-joint position to achieve a perfect superimposition of the medial and lateral posterior condyles. The standard lateral position for knee-joint radiography in males was found to be inwardly rotated at − 3.61° ± 6.91° and outwardly tilted at 2.65° ± 4.04°. In contrast, the female knee joint was inwardly rotated at − 3.61° ± 6.91° and outwardly tilted at 0.38° ± 3.06°. The optimized standard lateral position was employed in the clinical radiography of knee joints. The outward tilt angle was determined by the anatomical positional relationship of the upper end of the femur’s medial and lateral posterior condyles on the anteroposterior X-ray images. The inward rotation angle was determined by touch to define the anatomical positional relationship of the lower end of the femur’s medial and lateral posterior condyles. Our founding reveals that the application of this optimized standard lateral position was relatively effective, and the quality of the lateral knee-joint X-ray images captured in the new optimized position was generally higher compared with images captured in the conventional position. For most patients, the posterior condyles of the femoral epiphysis were in perfect superimposition. The optimized lateral knee-joint radiography position, obtained with the aid of knee-joint CT images and the MIP technique, was deemed to be efficient in clinical practice. For example, orthopedic doctors evaluated this method as significantly beneficial for the diagnosis of knee-joint conditions, for the assessment of the severity of the injury/condition, and for measurements of bone tissue before surgery (as in knee arthroplasty), thus ensuring the accuracy of the diagnosis and associated surgical operations.

There are certain limitations in this study. Due to the small sample size used, a significant deviation is introduced to our results. Herein, some trends were noted. For instance, some angles of inward rotation and outward tilt should be taken to the lateral knee joint for the acquisition of lateral knee-joint radiographs. This can provide some solutions for clinical requirements, such as the imaging requirement of perfect superimposition for posterior condyles of the femoral epiphysis and the position requirement of weight-bearing for patients. In the future, potential deviations in the sample cohort can be reduced with an increased sample size. This will also provide a more accurate representation of the efficacy of the optimized position.

In conclusion, the optimization of the knee-joint lateral radiography position provides orthopedic doctors and their patients with a better and more practical postural technique. This process is based on the conventional knee-joint lateral position that is inwardly rotated at − 3.61° ± 6.91° and − 3.61° ± 6.91° and outwardly tilted at 2.65° ± 4.04° and 0.38° ± 3.06° in the male and female knee joint, respectively. These findings also provide innovative guidance regarding the improvement in techniques related to the radiography position of human joints and introduces a novel direction for the application of CT image post-processing techniques.

## Data Availability

Data transparency: Yes.

## Abbreviations

CT: Computed tomography
MIP: Maximum intensity projection
SID: Source to image-receptor distance
FOV: Field of view
mSv: Millisievert
